# Periodontal tissue regeneration by transplantation of autologous adipose tissue-derived multi-lineage progenitor cells

**DOI:** 10.1038/s41598-022-11986-z

**Published:** 2022-05-17

**Authors:** Masahide Takedachi, Keigo Sawada, Kazuma Sakura, Chiaki Morimoto, Asae Hirai, Tomoaki Iwayama, Junpei Shimomura, Kohsuke Kawasaki, Chiharu Fujihara, Yoichiro Kashiwagi, Akimitsu Miyake, Tomomi Yamada, Hanayuki Okura, Akifumi Matsuyama, Masahiro Saito, Masahiro Kitamura, Shinya Murakami

**Affiliations:** 1 Department of Periodontology, Osaka University Graduate School of Dentistry, 1-8 Yamadaoka, Suita, Osaka 565-0871 Japan; 2 Department of Medical Innovation, Osaka University Hospital, Suita, Japan; 3 Center for Rare Disease Research, National Institute of Biomedical Innovation, Health and Nutrition, Ibaraki, Japan; 4 Department of Restorative Dentistry, Division of Operative Dentistry, Tohoku University Graduate School of Dentistry, Sendai, Japan

**Keywords:** Mesenchymal stem cells, Regeneration

## Abstract

Periodontitis is a chronic inflammatory disease that destroys tooth-supporting periodontal tissue. Current periodontal regenerative therapies have unsatisfactory efficacy; therefore, periodontal tissue engineering might be established by developing new cell-based therapies. In this study, we evaluated the safety and efficacy of adipose tissue-derived multi-lineage progenitor cells (ADMPC) autologous transplantation for periodontal tissue regeneration in humans. We conducted an open-label, single-arm exploratory phase I clinical study in which 12 periodontitis patients were transplanted with autologous ADMPCs isolated from subcutaneous adipose tissue. Each patient underwent flap surgery during which autologous ADMPCs were transplanted into the bone defect with a fibrin carrier material. Up to 36 weeks after transplantation, we performed a variety of clinical examinations including periodontal tissue inspection and standardized dental radiographic analysis. A 36-week follow-up demonstrated no severe transplantation-related adverse events in any cases. ADMPC transplantation reduced the probing pocket depth, improved the clinical attachment level, and induced neogenesis of alveolar bone. Therapeutic efficiency was observed in 2- or 3-walled vertical bone defects as well as more severe periodontal bone defects. These results suggest that autologous ADMPC transplantation might be an applicable therapy for severe periodontitis by inducing periodontal regeneration.

## Introduction

Periodontitis is a chronic inflammatory disease caused by bacterial biofilms formed on the root surface of teeth. This disease can lead to the irreversible destruction of periodontal tissue that includes alveolar bone, root cementum, periodontal ligaments, and gingiva. If left untreated, periodontitis impairs oral functions such as mastication and articulation, and eventually results in tooth loss^1,2^. Conventional treatment for periodontitis includes mechanical removal of the bacterial biofilm and debridement of necrotic cementum on the root surface and granulation tissue facing the periodontal pocket. This treatment can suppress the progression of periodontitis; however, it rarely regenerates the periodontal tissue to a clinically significant level^3^. Thus, such treatments need to be followed by subsequent surgical periodontal regeneration therapies including bone grafting, use of barrier membranes, and the topical application of enamel matrix derivatives or recombinant cytokines such as platelet-derived growth factor and basic fibroblast growth factor (FGF-2) to achieve successful treatment^4,5^. Unfortunately, indications for the existing periodontal regeneration therapies are limited to mild and moderate periodontal tissue destruction and clinical options to regenerate severe periodontal tissue destruction are limited.

Therefore, the development of therapeutic methods to induce periodontal tissue regeneration by the transplantation of mesenchymal stem cells (MSCs) is under intense investigation as a clinical approach for patients with severe periodontitis^4,6^. Transplantation of MSCs isolated from dental pulp and periodontal ligament, which can be collected in the dental field, have been examined in preclinical and clinical studies, highlighting their advantageous effects^7–10^. However, these treatments require an extractable wisdom tooth that is non-functional in the oral cavity and is not affected by a cavity or periodontitis. In the case of autologous transplantation, this requirement will significantly limit the number of indicated patients. However, MSCs can be isolated from bone marrow and adipose tissue. Sufficient amounts of subcutaneous adipose tissue can be obtained for MSC isolation using relatively easy and safe techniques. Furthermore, adipose tissue-derived MSCs have high proliferative ability and secrete a significant amount of trophic factors including growth factors^11,12^, indicating their potential and capability to stimulate tissue regeneration in transplantation therapy. As reported previously^13^, we established a method to isolate high-purity MSCs from adipose tissue. Cells harvested by treating the stromal vascular fraction with ethylenediaminetetraacetic acid were termed adipose tissue-derived multi-lineage progenitor cells (ADMPCs). ADMPCs demonstrated greater ability to differentiate into adipocytes, chondrocytes, and osteoblasts than adipose tissue-derived MSCs in vitro^14^. Furthermore, transplantation of ADMPCs, but not adipose tissue-derived MSCs, has been shown to be therapeutically effective in a disease model, indicating that the ADMPCs were high-purity MSCs^14^. We demonstrated the efficacy of the autologous transplantation of ADMPCs in periodontal tissue regeneration in preclinical studies using canine (beagle) experimental periodontitis models^15^. Furthermore, we reported that trophic factors secreted from ADMPCs stimulated the differentiation of periodontal ligament cells, a mode of action that is critical for periodontal tissue regeneration by cell-based therapies^16^.

In this study, we initiated a first-in-human open-label, single-arm exploratory phase I clinical study to assess the safety and efficacy of autologous ADMPC transplantation in periodontal tissue regeneration.

## Results

### Baseline characteristics of subjects

Consent for participation in this clinical study was obtained from 17 subjects, but after the screening test, three subjects violated the exclusion criteria, one patient withdrew consent during the study, and cell culture was discontinued for one patient because of contamination. Thus, autologous ADMPC transplantation was performed in 12 subjects. The mean (± SD) age of patients who were transplanted with ADMPCs was 53.25 ± 9.15 years (range, 43–72 years) when consent was initially obtained for this study. The types of teeth examined were two anterior teeth, one premolar tooth, and nine molars. There were five cases of one-wall, two cases of two-wall, and two cases of three-wall bone defects, and three cases of circumferential bone defects. The characteristics of each patient are presented in Table 1.

**Table 1:** Patient characteristics.

No	Sex	Age (years)	Tooth number	Defect shape	Defect position
1	F	44	33	3-wall	Distal-Buccal
2	F	51	27	Circumferential	Center-Buccal
3	M	72	36	1-wall	Mesial
4	F	66	17	1-wall	Distal
5	M	57	26	2-wall	Distal-Buccal
6	F	51	16	1-wall	Distal
7	F	44	16	1-wall	Mesial
8	F	58	17	Circumferential	Buccal
9	F	50	15	2-wall	Distal-Lingual
10	F	58	43	3-wall	Distal-Lingual
11	F	47	37	1-wall	Distal
12	F	43	47	Circumferential	Buccal

M, male; F, female.

### Cell characteristics and quality control of ADMPCs

**Figure 1:**
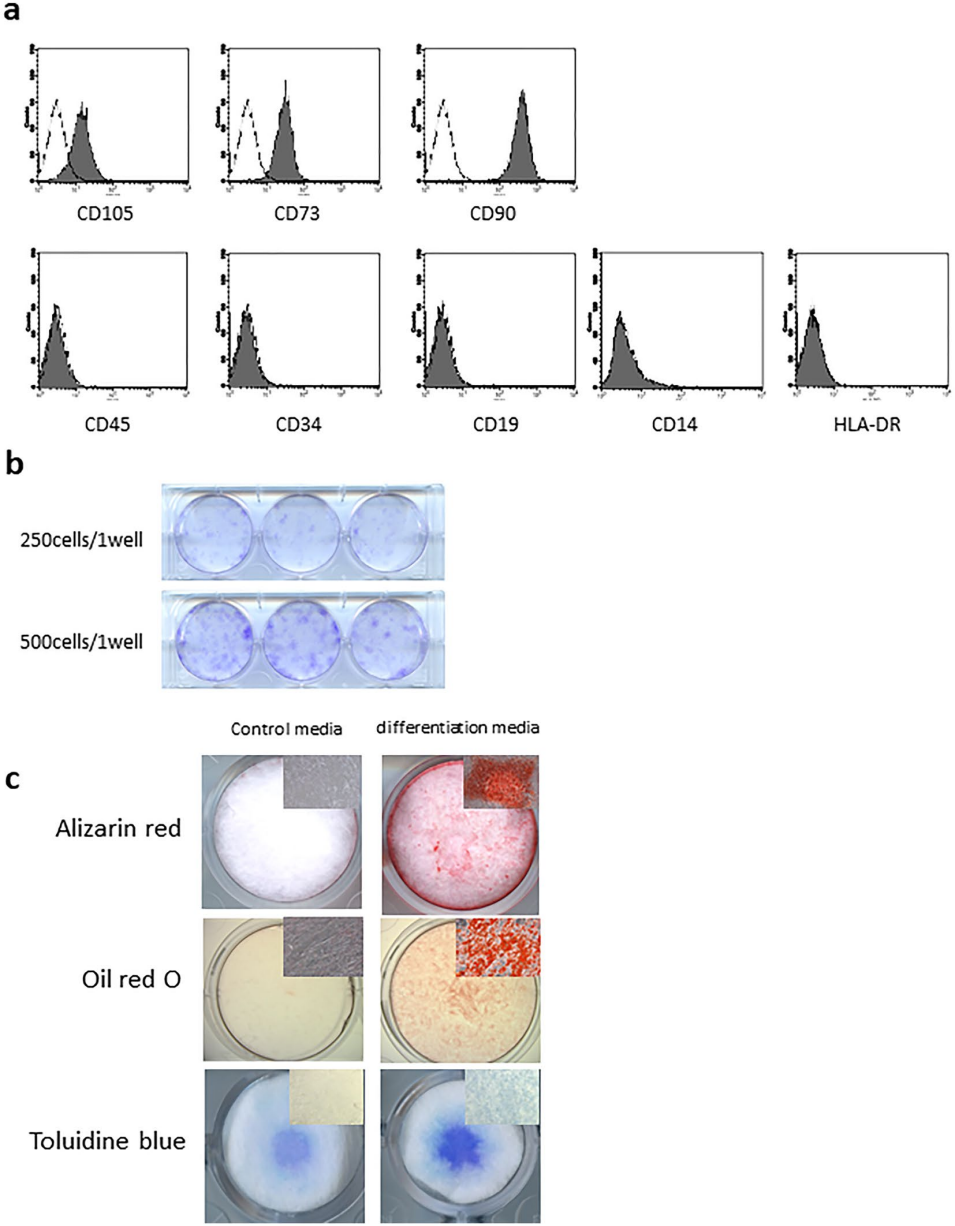
Characteristics of adipose tissue-derived multi-lineage progenitor cells (ADMPCs). (**a**) Mesenchymal stem cell (MSC) surface marker expression on ADMPCs. The expression of each surface marker is shown on the shaded histogram. Staining with an isotype control mAb is represented by a black line. (**b**) Colony formation of ADMPCs. Cells were seeded with the indicated density and cultured for 2 weeks. Cells were then fixed and stained with 0.1% crystal violet. (**c**) Multipotency of ADMPCs. Representative images are shown of Alizarin red staining of ADMPCs cultured in osteogenic differentiation media for 28 days, Oil red O staining of ADMPCs cultured in adipogenic differentiation media for 24 days, and Toluidine blue staining of ADMPCs cultured in chondrogenic differentiation media for 20 days.

Flow cytometry analysis demonstrated the positive expression of CD105, CD73, and CD90, and lack of CD45, CD34, CD19, CD14, and HLA-DR (Fig. 1a) on ADMPCs. Additionally, ADMPCs demonstrated colony formation (Fig. 1b) and multipotent differentiation to osteoblasts, adipocytes, and chondrocytes (Fig. 1c). Thus, the ADMPCs used in this study are consistent with the definition of MSCs^17^. As a quality control, cell number, viability, and purity were measured before transplantation and each value had cleared the pre-specified threshold (Supplemental Table 1). Results from the infection tests, including a sterility test, mycoplasma negative test, and endotoxin test, indicated no bacterial infection in any transplanted ADMPCs.

### Safety evaluation

**Figure 2:**
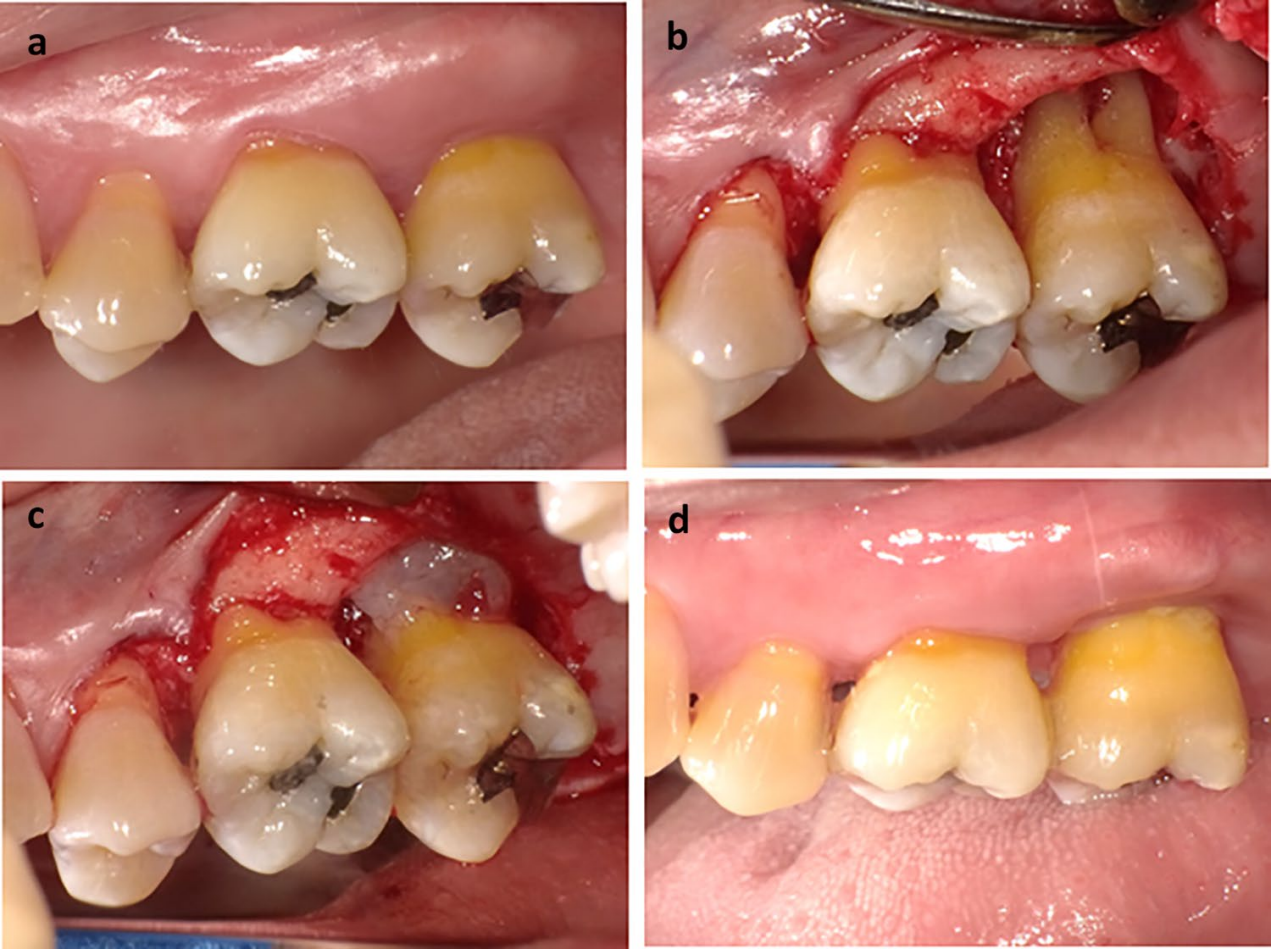
Representative images of adipose tissue-derived multi-lineage progenitor cell (ADMPC) transplantation. (**a**) Pre-operative intra-oral findings. This case was a 51-year-old female. The test tooth was an upper left second molar with a 6-mm periodontal pocket in the distal area. (**b**, **c**) Intra-oral findings during periodontal surgery. The flap operation was performed in accordance with the modified Widman procedure. After reversing the gingival flap, a circumferential intra-bony defect filled with granulation tissue was revealed and the granulation tissue was removed. After washing the intra-bony defect with normal saline, ADMPCs mixed with fibrin gel were transplanted into the intra-bony defect. (**d**) Postoperative intra-oral findings. Four weeks after ADMPC transplantation, no abnormal findings were found at the surgical site.

All ADMPC transplantation procedures (Fig. 2) were performed without problems. During the follow-up period, several adverse events for which a causal relationship could not be ruled out were observed (Table 2). Results from urine tests and blood tests are shown in Supplemental Tables 2 and 3. No changes considered clinically important were observed including high C-reactive protein, which was observed in 2 of 12 patients at 9 months after transplantation.

**Table 2:** Adverse events.

Adverse events	Severity (N = 12)		
	Grade 1	Grade 2	Grade 3
Pain at the site of cell transplantation within a week of transplantation	2 (16.7%)	10 (83.3%)	–
Discomfort at the site of cell transplantation after more than a week of transplantation	3 (25.0%)	–	–
C-reactive protein increased	2 (16.7%)	–	–
Temporary inflammation of gingival tissue	1 (8.3%)	1 (8.3%)	–
Angular stomatitis	–	1 (8.3%)	–
Occlusal pain	–	1 (8.3%)	–
Stomatitis	1 (8.3%)	–	–
Temporary necrosis of gingival epithelium	1 (8.3%)	–	–
Depression of gingival tissue after surgery	1 (8.3%)	–	–

Grade 1: Mild, no treatment or treatment with medical material or quasi-drugs was required but possible to continue the study, Grade 2: Moderate, treatment with medicine was required but possible to continue the study, Grade 3: Severe, unable to continue the study.

Although postoperative symptoms in the oral cavity, including temporary pain, were observed at the surgical site in all patients, these disappeared within 1 week. Additionally, angular inflammation of the mouth in 2 of 12 patients and postoperative dentin hypersensitivity and delayed wound healing in 1 of 12 patients were noted.

### Efficacy evaluation

**Figure 3:**
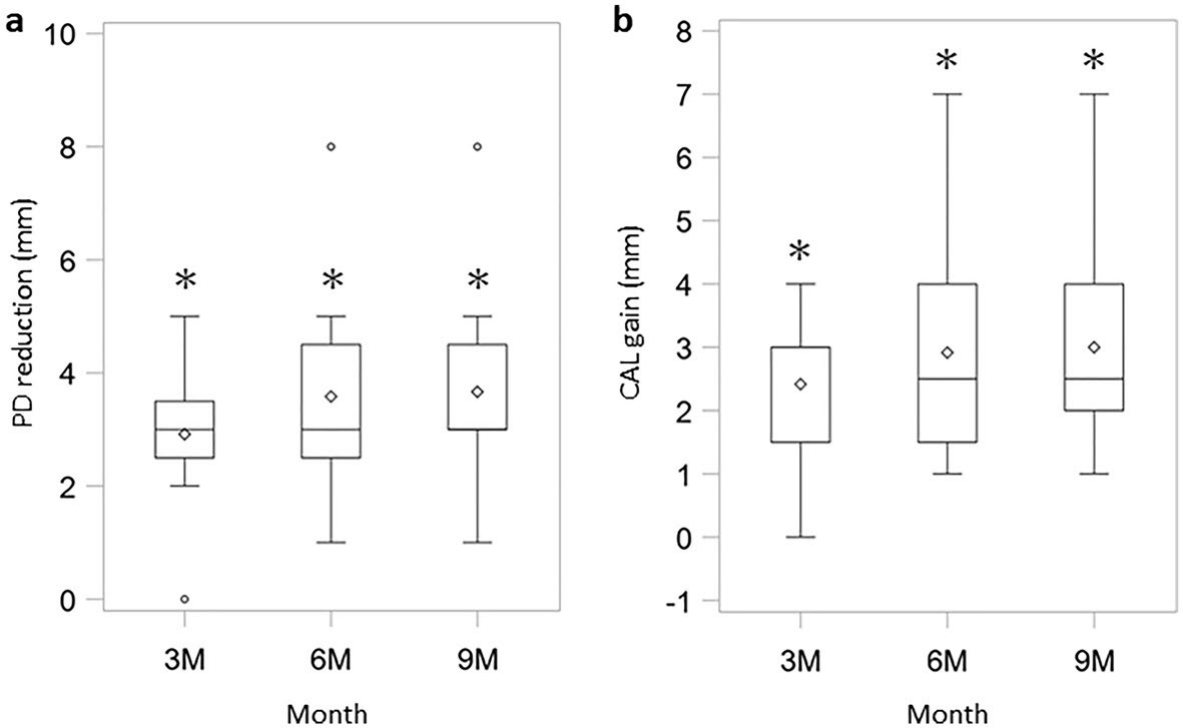
Clinical assessment of probing pocket depth (PD) and clinical attachment level (CAL). Results of (**a**) PD reduction and (**b**) CAL gain are shown as box-and-whisker plots. The box, notch, and horizontal line denote the distance between the first and third quartile ranges (interquartile range; IQR), mean, and median, respectively. The upper whisker indicates a maximum value smaller than 1.5 × IQR above the third quartile (Q3). Similarly, the lower whisker indicates a minimum value greater than 1.5 × IQR below the first quartile (Q1). It also displays the outliers which were defined as further outside than Q1 − 1.5 × IQR or Q3 + 1.5 × IQR. PD reduction and CAL gain of the test sites were measured at 3 months, 6 months, 9 months (n = 12). Individual data is shown in Supplemental Table 4. *p < 0.0001, with the use of a one-sample *t*-test based on the closed testing procedure.

Assessment of the efficacy of the regeneration therapy was determined by changes in probing pocket depth (PD) and clinical attachment level (CAL) gain (Fig. 3, Supplemental Table 4). ADMPC transplantation decreased the PD and acquisition of CAL over time. The PD was 2.92 ± 1.24 mm after 12 weeks, 3.58 ± 1.83 mm after 24 weeks, and 3.67 ± 1.78 mm after 36 weeks. Statistical analysis showed a significant reduction in the PD from 12 to 36 weeks after transplantation. Gain of CAL was 2.42 ± 1.24 mm at 12 weeks after transplantation, 2.92 ± 1.83 mm at 24 weeks after transplantation, and 3.00 ± 1.76 mm at 36 weeks after transplantation. Statistical analysis showed a significant gain in CAL from 12 to 36 weeks after ADMPC transplantation.

**Figure 4:**
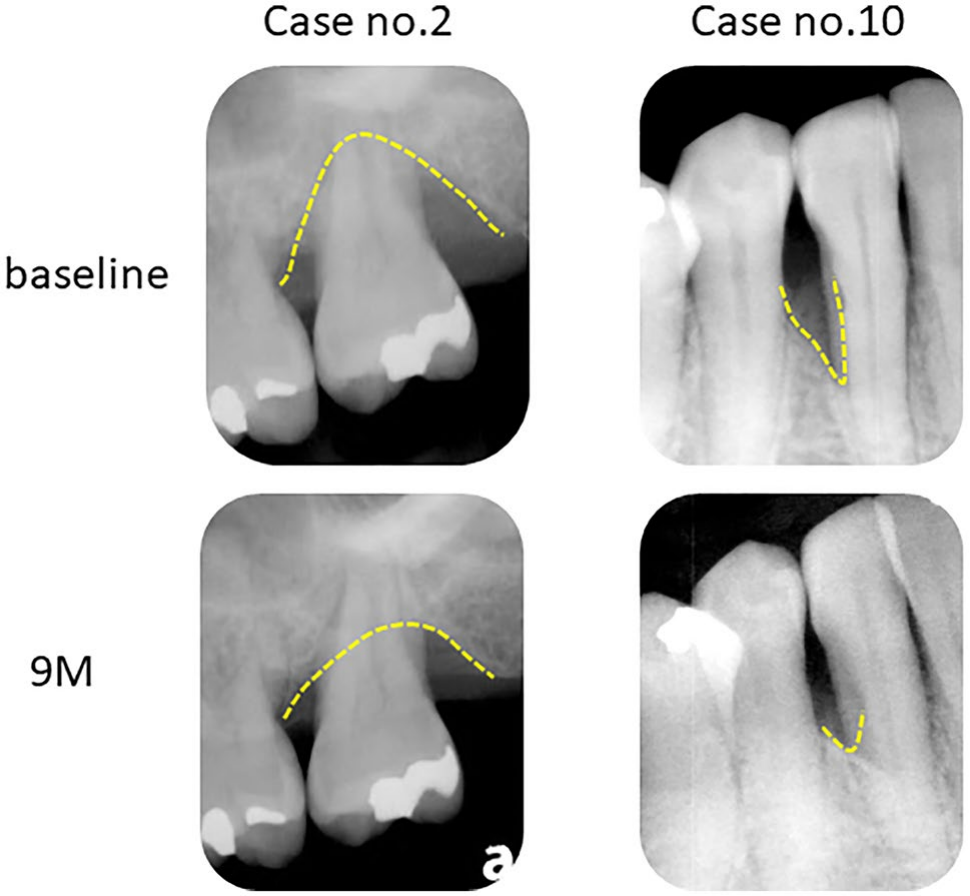
Outcome of adipose tissue-derived multi-lineage progenitor cell (ADMPC) transplantation by dental radiographs. Radiographic outcomes of ADMPC-transplanted individuals. Case no. 2 was a 51-year-old woman. Case no. 10 was a 58-year-old woman. Dotted lines indicate the remaining alveolar bone crest or the bottom of the bone defect. The radiographs clearly show that the bone defect was filled with newly generated alveolar bone at 9 months after transplantation.

**Figure 5:**
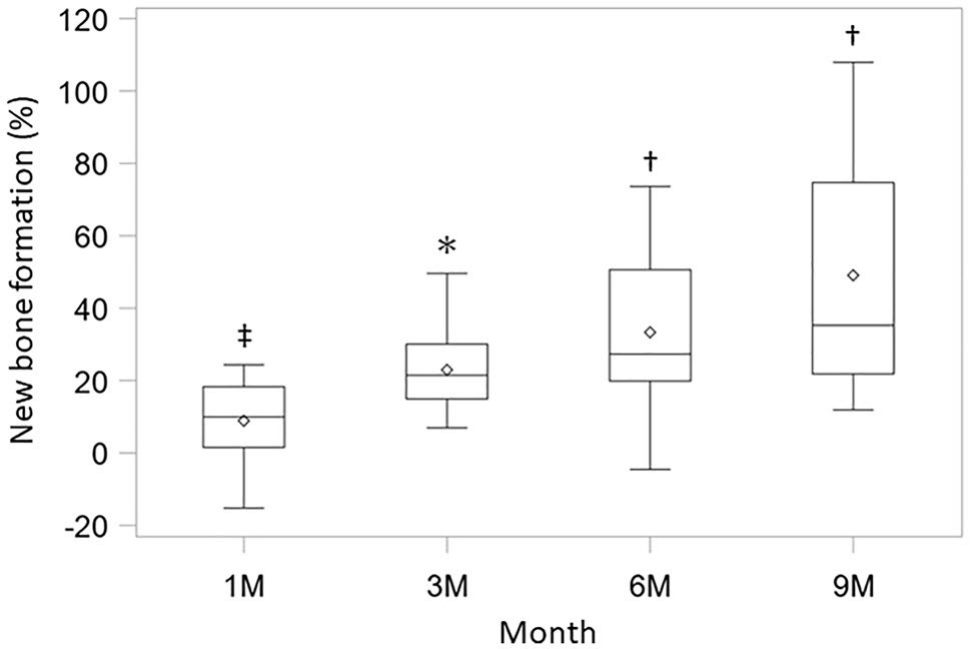
Clinical assessment of new bone formation. Results of new bone formation are shown using the same box-and-whisker plot representation as in Fig. 2. The new bone formation rate was measured using X-ray images at 1 month, 3 months, 6 months, and 9 months (n = 12). *p < 0.0001, ^†^*p* < 0.001, ^‡^*p* < 0.025, with the use of a one-sample *t*-test based on the closed testing procedure.

Representative radiographs of ADMPC-transplanted participants are shown in Fig. 4. The rate of increase in new alveolar bone was 8.93 ± 11.54% after 4 weeks, 22.98 ± 12.57% after 12 weeks, 33.36 ± 23.91% after 24 weeks, and 49.13 ± 32.16% after 36 weeks, with a significant increase over time (Fig. 5).

The number of bleeding on probing-positive sites decreased over time. Patient-level analysis showed a significant decrease over time, with 11 patients positive at baseline, 4 at 12 weeks, 1 at 24 weeks, and 1 at 36 weeks. The gingival index showed an improvement of 83.3% (95% confidence interval: 51.6–97.9) in the patient-level analysis compared with baseline, with 10 of 12 patients showing an improvement at 36 weeks, and 2 of 12 patients scoring the same as baseline (gingival index score: 0).

**Table 3:** Schedule of the clinical study.

	Screening (before registration)	At the time of transplant	After transplantation					
			1 wk	2 wk	1 M	3 M	6 M	9 M
Observation of general condition	●	●	●	●	●	●	●	●
Observation of the oral cavity	●	●	●	●	●	●	●	●
Observation of liposuction site		●			●	●		●
Blood test	●	●	●		●	●		●
Urine test	●	●	●		●	●		●
12-lead electrocardiogram	●				●			●
Chest radiograph	●				●			●
Dental radiograph	●	●			●	●	●	●
Periodontal examination	●	●				●	●	●

## Discussion

In the field of periodontology, the development of therapeutic methods has been driven forward to achieve the ideal goal of anatomical and functional periodontal tissue regeneration for severe periodontal disease, because existing periodontal tissue regeneration therapies are not expected to be sufficiently effective. Recently, the rapid growth of stem cell biology has accelerated the development of cell-based therapy for periodontal tissue regeneration^6^. While the effectiveness of therapeutic methods that require the sacrifice of healthy teeth for the isolation of stem cells such as dental pulp stem cells and periodontal ligament stem cells has been clinically evaluated^8–10^, treatments using mesenchymal stem cells isolated from the bone marrow or adipose tissue as non-tooth-derived cells have been limited to preclinical studies or case reports^18,19^. To the best of our knowledge, this is the first clinical study on the use of MSCs from adipose tissue for the regenerative treatment of periodontal tissue.

Regarding safety, which was the primary endpoint in this clinical study, all observed adverse events were those that have already been reported for periodontal surgery including existing periodontal tissue regeneration therapy. Additionally, these adverse events did not affect the efficacy of the treatment and were not considered related to ADMPC transplantation^20^. We collected 10–30 mL of subcutaneous adipose tissue, which is relatively easy to collect from adults. This was sufficient to isolate ADMPCs. However, to ensure safety in this study, one lean subject was excluded after screening indicated they lacked sufficient subcutaneous adipose tissue mass. In terms of the safety of liposuction, 8 of the 12 subjects had discomfort at the site of the adipose tissue extraction, which resolved within 4 weeks of surgery. No infections or contour irregularities at the surgical site, which are generally reported as side effects of subcutaneous adipose tissue harvesting, were observed^21^. Furthermore, no subject refused to participate in the study because of the aspiration of subcutaneous adipose tissue, which indicated that this treatment is acceptable for periodontitis patients.

The recent guideline indicated that the safety and quality of animal-derived materials represented by fetal bovine serum used in cell-based therapy should be fully verified^22^. In this study, we used fetal bovine serum native to Australia, which is free from bovine spongiform encephalopathy in addition to mycoplasma and 9CFR viruses. This was approved as a result of a review in accordance with the Japanese Standards for Biological Ingredients at the time when the research protocol was approved. In future, when conducting a phase II clinical trial with an increased number of subjects, it will be necessary to establish a cell culture process in accordance with the latest guidelines and the laws of the country of implementation, which will eventually lead to the provision of safer cell therapy using ADMPCs.

The ratio of alveolar bone regeneration (49.13 ± 32.16%) at 36 weeks after transplantation was related to ADMPCs because the fibrin gel we used as a scaffold is not radiopaque or osteoinductive. Together with the gain of CAL (3.00 ± 1.76 mm), these efficacy evaluation indexes were comparable to those obtained in clinical trials in which FGF-2 was investigated for periodontal tissue regeneration (bone fill ratio: 34.369–58.62%, CAL gain: 2.1–2.7 mm)^23–25^. Of note, the target subjects in the clinical trials that evaluated FGF-2 were periodontitis patients with 2- or 3-walled vertical periodontal tissue defects. Subjects in the current study included those with more severe periodontal tissue defects, which indicates that the efficacy observed after ADMPC transplantation was clinically significant.

All studies that have reported cell-based periodontal tissue regeneration therapies to date have evaluated cell transplantation in combination with β-TCP and collagen sponge, which have osteoinductive abilities, or non-resorbable bone substitute derived from bovine bone (Bio-oss®) or guided tissue regeneration (GTR) membrane. For example, Chen et al.^10^ investigated the potential additional effects of periodontal ligament stem cells in combination with Bio-oss® and GTR membrane in a clinical study. Because the target cases had moderate periodontitis and sufficient periodontal tissue regeneration occurred in the controls, the effects of cell transplantation were not demonstrated. Of note, Iwata et al.^9^ reported the combined effects of β-TCP and periodontal ligament cell sheets, and demonstrated the efficacy of cell therapy in comparison with the results of previously performed clinical trials in which β-TCP was administered^26^. In the current study, we could not set a control group from an ethical perspective. However, the effectiveness we observed in severe periodontal tissue defects including 1-walled and circumferential bone defects demonstrated clinical importance, even though we used fibrin gel as a scaffold, which is not expected to regenerate periodontal tissue. In a future study, we will select a scaffold material that can support or enhance the proliferation, differentiation, and trophic factor secretion of ADMPCs, which might stimulate further periodontal tissue regeneration by ADMPC transplantation. In addition, the number of transplanted cells is an issue yet to be determined. In this study, the dose of ADMPC was set to contain as many cells as possible within the range in which the hardening of the fibrin gel could be confirmed. When changing the scaffold material, it might be necessary to reconsider the relationship between cell number and the therapeutic effects, in addition to the physical characteristics and operability of the ADMPC-scaffold complex.

MSC transplantation has been reported to have effects on various diseases^27^. Allogeneic transplantation is an option when MSC-derived trophic factors contribute to healing and tissue regeneration because the immunomodulatory activity of MSCs should minimize immune rejection after transplantation. Periodontal tissue regeneration is no exception, and one case report demonstrated the effect of allogeneic mesenchymal dental pulp stem cells^28^. We previously demonstrated that trophic factors from ADMPCs stimulate the differentiation of periodontal ligament cells^16^. Therefore, we examined the efficacy of allogeneic transplantation of ADMPCs for periodontal tissue regeneration and reported findings in animal studies where allogeneic transplantation of ADMPCs promoted periodontal tissue regeneration without inducing a severe inflammatory response^29^. If treatment by allogeneic cell transplantation is established, the quality of the cell preparation can be more stable, batch-to-batch variation can be avoided, and a more stable therapeutic effect can be expected. Induced pluripotent stem cells are expected to provide a source of stable MSCs^30,31^, and a recent report demonstrated the therapeutic effects on graft-versus-host disease by administration of GMP-grade MSCs derived from human induced pluripotent stem cells as a clinical trial^32^. The progress of such research raises expectations for its application in many fields including periodontal tissue regeneration. However, after transplantation of allogeneic cells into periodontal tissue, antibody production in response to the allogeneic cells was observed, which was not seen in autologous transplantation in our preclinical study^29^. Unlike diseases in which a single MSC administration is sufficiently effective, patients who require periodontal tissue regeneration may require separate operations at different sites. It is expected that the second and third allogeneic transplantation will increase the immunogenicity of the transplanted cells; therefore, stringent safety verification will be required to proceed with the development of allograft therapy.

## Conclusion

In this study, we showed that autologous ADMPC transplantation to the local site of periodontal destruction was safe and effective in tissue regeneration.

## Methods

### Study design

An open-label, single-arm exploratory phase I study was conducted in accordance with the principles expressed in the Declaration of Helsinki, the guidelines on Clinical Stem Cell Research from the Ministry of Health, Labour and Welfare, and the Act on the Safety of Regenerative Medicine. The protocol was reviewed and approved by the ethical review committee at Osaka University Dental Hospital (H21-E30) and the First Certified Special Committee for Regenerative Medicine, Osaka University (PB5150004). This clinical study was registered with the UMIN Clinical Trials Registry (No. UMIN000007698, 9th April 2012) and performed in Osaka University Dental Hospital. This study included 17 patients with severe periodontitis who had a 7-mm or deeper periodontal pocket depth at the initial visit. After a standard initial preparation, including oral hygiene instruction, full-mouth scaling and root planing, regions of investigation were determined as vertical periodontal tissue defects (depth ≥ 4 mm and width ≥ 2 mm) using dental radiographs and periodontal tissue inspection. Screening tests were performed before registration to see if each patient met the criteria. A list of all inclusion/exclusion criteria is shown in Supporting Information Appendix S1.

### ADMPC preparation

After the administration of local anesthesia with an anesthetic (10 mL 2% lidocaine hydrochloride, 1 mL 7% sodium hydrogen carbonate, and 30 mL normal saline), subcutaneous fatty tissue was extirpated from periodontitis patients by liposuction with a tulip cannula (Kakinuma Medical Inc. Tokyo, Japan). ADMPCs were isolated and cultured as described previously^33^. Briefly, aspirated adipose tissue was washed three times with wash buffer (60 mg/mL kanamycin in phosphate buffered saline; PBS) and then digested at 37 °C for 1 h in 0.083% collagenase (Wako Pure Chemical Industries, Osaka, Japan) in PBS. Digested tissue was diluted by adding Dulbecco’s modified Eagle’s medium–high glucose (DMEM-HG; Thermo Fisher Scientific, Waltham, MA, USA) with 20% fetal bovine serum (FBS; Moregate BioTech, Bulimba, Australia, Lot #27,301,113) and centrifuged at 400×*g* for 10 min. The cell pellet was suspended with DMEM-HG with 10% FBS and red blood cells were excluded using density gradient centrifugation with Histopaque (d = 1.077 g/mL; Sigma-Aldrich, St. Louis, MO, USA). Cells were then filtered through a 40-µm cell strainer and cultured in DMEM-HG with 10% FBS at 37 °C for 24 h. Following incubation, cells were washed with wash buffer and treated with 0.02% ethylenediaminetetraacetic acid solution (Nacalai Tesque, Kyoto, Japan). Floating cells, termed ADMPCs, were collected and seeded onto a fibronectin-coated dish (BD Biosciences, San Jose, CA, USA) in medium comprising 60% DMEM-low glucose (Thermo Fisher Scientific), 40% MCDB-201 medium (Sigma-Aldrich), 1 nM dexamethasone (Sigma-Aldrich), 100 μM L-ascorbic acid (Sigma-Aldrich), 10 mg/L insulin-transferrin-selenium solution (Thermo Fisher Scientific), 10 ng/mL epidermal growth factor (PeproTech, Rocky Hill, NJ, USA), 60 μg/mL kanamycin, and 5% FBS. After two passages, ADMPCs were frozen in 20% FBS and 10% dimethyl sulfoxide in DMEM- low glucose. Fourteen days before transplantation, ADMPCs were thawed and cultured for two passages. All cell culture steps were performed in a cell processing isolator (Shibuya Corporation, Kanazawa, Japan).

FBS was selected in compliance with the Japanese Standards for Biological Ingredients and all reagents used to culture ADMPCs were approved by the ethical review committee at Osaka University Dental Hospital and the First Certified Special Committee for Regenerative Medicine, Osaka University.

### Characterization of ADMPCs

Cell surface markers on ADMPCs were analyzed by flow cytometry using FITC-labeled anti-CD105, anti-CD73, anti-CD90, anti-CD45, anti-CD34, anti-CD14, anti-CD19, and anti-HLA-DR antibodies and isotype control antibody (BD Biosciences). Data were collected with a FACSCalibur (BD Biosciences). A colony forming assay was performed as previously described^34^. Briefly, 250 or 500 ADMPCs were seeded on a 6-well plate and cultured for 2 weeks. Cells were washed with PBS followed by fixation with 4% paraformaldehyde solution and staining with 0.1% crystal violet solution (Muto Pure Chemicals Co. Ltd, Tokyo, Japan). The multipotency of ADMPCs was assessed by cytodifferentiation to osteoblasts, adipocytes and chondrocytes. To induce osteogenic differentiation, ADMPCs were cultured with DMEM-HG supplemented with 10% FBS, 10 mM β-glycerophosphate (Wako Pure Chemical Industries) and 50 µg/mL ascorbic acid (Wako Pure Chemical Industries) and 0.1 µM dexamethasone (Sigma-Aldrich) for 28 days. For adipogenic differentiation, ADMPCs were cultured with DMEM-HG supplemented with 10% FBS, 200 µM indomethacin (Sigma-Aldrich), 500 µM 3-isobre-1-methylxanthine (Sigma-Aldrich) and 1 µM dexamethasone (Sigma-Aldrich) for 24 days. Chondrogenic differentiation was induced as previously described^35^. Briefly, ADMPCs were harvested and suspended in chondrocyte growth medium (PromoCell, Heidelberg, Germany) at 2 × 107 cells/mL, and 12.5 µL droplets were placed in the center of each well of a 24-well plate. Cells were allowed to adhere for 2 h, followed by the addition of 500 µL of chondrocyte growth medium with SupplementMix (PromoCell). In each culture condition, the medium was replaced every 3 days. The ability of ADMPCs to differentiation into osteoblasts, adipocytes, and chondrocytes was evaluated by Alizarin red S, Oil red O, and Toluidine blue staining, respectively.

### Quality control of ADMPCs for transplantation

ADMPCs were examined for quality at the time of cell freezing, cell thawing, and just before transplantation. Quality control tests included cell number, cell viability, cell purity, and infection tests. Cell number and viability were determined by staining with 0.4% (w/v) Trypan blue solution (Wako Pure Chemical Industries). Cell purity was analyzed by flow cytometry using FITC-labeled anti-CD45 antibody (BD Biosciences), FITC-labeled anti-CD105 antibody (Ancell Corporation, Bayport, MN, USA), FITC-labeled anti-CD166 antibody (Ancell Corporation), and FITC-labeled isotype control mouse IgG1 antibody (BD Biosciences). Data were collected with a FACSCalibur (BD Biosciences). The culture supernatant was collected by aspiration and used for infection tests including a sterility test, mycoplasma negative test, and endotoxin test.

### ADMPC transplantation

The flap operation was performed in accordance with the modified Widman procedure. After the induction of local infiltrated anesthesia with 2% lidocaine hydrochloride and 1/80,000 epinephrine, full-thickness buccal and lingual flaps were elevated. Granulation tissue around the root was removed with subgingival soft and hard deposits on the root surface using hand and ultrasonic instrumentation. After ensuring all quality control tests were passed, we mixed ADMPCs diluted 1:6 with fibrin gel (Beriplast® P Combi-Set tissue adhesive; CSL Behring K.K., Tokyo, Japan or Bolheal®; Teijin Pharma Limited, Tokyo, Japan) to prepare an ADMPC-fibrin gel complex (4.2 × 10^7^ cells/mL). The bone defect was filled with the ADMPC-fibrin gel complex without any specific root conditioning (Fig. 2). Then, surgical closure was performed. One operator (M.K.) performed all the surgical procedures.

### Outcome measurements

The primary outcome was the safety of this treatment. We examined whether and to what extent adverse events emerged that had causal relationships with ADMPC transplantation. Clinical findings for the oral cavity and the whole body were examined by interview and visual inspection in addition to several clinical inspections including analysis of blood and urine from 1 week to 9 months after transplantation. Adverse event severity was assessed using three grades: grade 1—mild, no treatment or treatment with medical material or quasi-drugs was required but possible to continue the study; grade 2—moderate, treatment with medicine was required but possible to continue the study; and grade 3—severe, unable to continue the study.

The secondary outcome was the efficacy of the treatment. PD, CAL, bleeding on probing, gingival index, and the percentage of bone fill were evaluated by standardized radiographs at baseline and at 3, 6, and 9 months. Geometrically standardized radiography used photographic indicators (Cone Indicator-II; Hanshin Technical Laboratory, Nishinomiya, Japan) and the rate of increase in alveolar bone height was calculated using the methods described previously^23^. The schedule of this clinical study is shown in Table 3.

Each outcome was assessed as changes from the baseline obtained at the time of ADMPCs transplantation.

### Statistical analysis

Continuous variables were presented as descriptive statistics (mean and standard deviation [SD]) and categorical variables were summarized as counts and percentages. With respect to the rate or amount of change of PD, CAL, and bone fill, *t*-tests based on a hierarchical closed-testing procedure were performed to control the overall type I error rate. Using this procedure, the rate or amount of change at each time point was tested using a one-sample *t*-test from 36 weeks post-transplantation to 4 weeks post-transplantation in order. The null hypothesis was that the change from baseline was zero. Hierarchical closed testing was continued until the last testing for which a *p* value was non-significant. The Clopper–Pearson exact method was used to estimate the gingival index improvement rate and its exact 95% confidence interval. One-sided *p*-values of less than 0.025 were considered to indicate statistical significance. Analyses were performed using SAS software, version 9.4 (SAS Institute, Cary, NC, USA).

### Ethics approval and consent to participate

The protocol was reviewed and approved by the ethical review committee at Osaka University Dental Hospital (H21-E30) and the First Certified Special Committee for Regenerative Medicine, Osaka University (PB5150004). All patients provided written informed consent to participate in the study.

### Consent for publication

All patients gave a consent for their clinical data to be used for publication.

## Supplementary Information

Supplementary Information.

## Data Availability

The data that support the findings of this study are available on request from the corresponding author.
