# Supplementary material for: Periodontal tissue regeneration by transplantation of autologous adipose tissue-derived multi-lineage progenitor cells
Source: Sci Rep. 2022 May 17;12:8126. doi: 10.1038/s41598-022-11986-z (PMC9114023; doi:10.1038/s41598-022-11986-z)
Supplement: Supplementary file 1 — Supplementary Information. [file 41598_2022_11986_MOESM1_ESM.pdf]

Supporting Information Appendix S1

Inclusion and exclusion criteria

Inclusion criteria

Patients with a probing depth of 7 mm or more on the first visit.

Patients with a vertical bone defect in the interdental area (including either mesial or distal of the test tooth) that is at least 4-mm deep and 2-mm wide as determined by radiographic evaluation.

Patients who have completed the initial periodontal treatment at the time of reassessment.

Patients with less than 2° of mobility of the test tooth and the presence of keratinized gingiva for which flap surgery is indicated.

Patients with stable oral hygiene at the start of the study and after stem cell transplantation.

Patients over 20 years of age at the time of obtaining consent for the study.

Patients who agreed to participate in this clinical study.

Exclusion criteria

Patients with prosthetics or other devices that interfere with the accurate measurement of clinical attachment levels.

Patients with concomitant or pre-existing malignancy.

Patients with findings of malignancy or precancerous lesions in the oral cavity or suspected findings.

Patients who have used or will use bisphosphonates.

Patients in need of a procedure (surgical or prosthetic procedure) that will affect the evaluation of the site under this study.

Patients who are pregnant, breastfeeding, seeking to become pregnant, or who may become pregnant.

Patients with concomitant renal, hepatic or blood disorders.

Patients with a hemoglobin A1c of at least 6.5% on pre-registration clinical examination.

Patients with an active infection.

Patients with a history of alcoholism or drug addiction within six months prior to enrollment.

Patients with a psychiatric complication or presenting with symptoms of a psychiatric disorder.

Patients with positive HCV, HBs, ATLA, or HIV antibodies.

Patients who are deemed by the principal investigator to be inappropriate for participation in the study.

Supplemental Table.1 Cell number, viability and purity of transplanted ADMPC

n=12		Freezing	Thawing	Transplantation
Cell number(x10^6)		7.2±2.4	-	16.9±9.7
		3.0		6.7
Cell viability (%)		96.4±1.3	89.1±4.3	94.2±3.1
		70	50	70
Purity	CD45 negative (%)	99.2±0.4	-	99.2±0.6
		70		70
	CD105 positive (%)	96.0±4.1	-	96.8±1.3
		70		70
	CD166 positive (%)	92.2±4.9	-	96.3±1.3
		70		70

Upper column indicates data (mean±SD), lower column indicates minimum threshold.

Supplemental Table2. Urine tests.

Urine protein (normal: -)

No	Baseline	1W	1M	3M	9M
1	−	−	−	−	+
2	−	−	−	−	−
3	−	−	−	−	−
4	−	−	−	−	−
5	−	−	−	−	−
6	−	−	−	−	−
7	−	−	−	−	−
8	−	−	−	−	−
9	−	−	−	−	−
10	−	−	−	−	−
11	−	−	−	−	−
12	−	a	−	−	−

a; missing value due to patient matter

Urine sugar (normal: ー)

No	Baseline	1W	1M	3M	9M
1	ー	ー	ー	ー	ー
2	ー	ー	ー	ー	ー
3	ー	ー	ー	ー	ー
4	ー	ー	ー	ー	ー
5	ー	ー	ー	ー	ー
6	ー	ー	ー	ー	ー
7	ー	ー	ー	ー	ー
8	ー	ー	ー	ー	ー
9	ー	ー	ー	ー	ー
10	ー	ー	ー	ー	ー
11	ー	ー	ー	ー	ー
12	ー	a	ー	ー	ー

a; missing value due to patient matter

Urobilinogen（EU/*dL*）(normal range: ≤1.0)

No	Baseline	1W	1M	3M	9M
1	0.1	0.1	0.1	0.1	1
2	0.1	0.1	0.1	0.1	0.1
3	0.1	0.1	0.1	0.1	0.1
4	0.1	0.1	0.1	0.1	0.1
5	0.1	0.1	0.1	0.1	0.1
6	0.1	0.1	0.1	0.1	0.1
7	0.1	0.1	0.1	0.1	0.1
8	0.1	0.1	0.1	0.1	0.1
9	0.1	0.1	0.1	0.1	0.1
10	0.1	0.1	0.1	0.1	0.1
11	0.1	0.1	0.1	0.1	0.1
12	0.1	a	0.1	0.1	0.1

a; missing value due to patient matter

Urine occult blood (normal: -)

No	Baseline	1W	1M	3M	9M
1	−	−	−	−	+++
2	−	−	−	±	−
3	−	−	−	+++	+
4	+	±	+	+	+
5	−	+	±	+	±
6	−	±	±	−	+
7	+	+	+	++	+
8	+	+	+	+	+
9	++	±	++	+	+
10	−	−	−	−	−
11	±	±	±	±	−
12	±	A	−	−	−

a; missing value due to patient matter

Supplemental table 3. Blood tests.

Basophil (%) (normal range: 0-1)

No	Baseline	1W	1M	3M	9M
1	0.9	0.9	0.8	0.9	0.8
2	0.5	0.5	0.6	0.7	0.8
3	0.4	0.5	0.4	0.5	0.5
4	0.8	0.9	0.6	0.6	0.5
5	0.5	0.4	1	0.9	0.5
6	1.1	1	0.8	0.9	0.9
7	0.9	0.6	0.9	0.8	0.4
8	0.4	0.4	0.2	0.2	0.2
9	0.9	0.8	0.6	0.5	0.8
10	0.4	1	0.7	0.3	0.2
11	1.4	1.3	1.4	1.2	0.9
12	0.3	0.4	0.5	0.4	0.4

Neutrophil (%) (normal range: 40-72)

No	Baseline	1W	1M	3M	9M
1	63.5	60.5	65.4	64.5	64.5
2	65.4	55.3	55.1	57.9	63.0
3	62.9	52.3	52.2	49.5	65.7
4	57.4	59.1	54.9	67.8	58.2
5	49.1	57.9	50.1	54.2	59.1
6	55.3	51.2	56.2	47.2	47.1
7	72.7	76.6	74.6	71.9	73.0
8	45.1	47.4	60.8	53.6	56.3
9	54.8	54.5	56.3	61.9	56.4
10	70.1	73.0	72.6	63.4	66.5
11	58.1	60.8	55.5	64.2	63.8
12	53.4	44.0	53.8	57.3	49.4

Eosinophil (%) (normal range: 0-7)

No	Baseline	1W	1M	3M	9M
1	3.1	2.7	2.0	3.0	2.8
2	3.2	4.2	3.4	2.9	2.8
3	2.0	2.8	2.0	3.3	2.1
4	3.0	3.7	3.4	2.9	3.7
5	2.7	2.9	2.4	3.4	3.1
6	3.8	2.5	2.6	3.6	1.6
7	1.0	1.2	1.9	1.0	1.1
8	1.3	1.9	1.0	2.9	1.0
9	2.7	2.3	2.3	1.9	3.6
10	2.5	2.5	2.4	2.4	1.8
11	4.0	3.3	5.0	2.1	2.2
12	4.2	6.6	4.2	5.2	2.6

Monocyte (%) (normal range: 0-12)

No	Baseline	1W	1M	3M	9M
1	7.5	6.9	7.0	6.0	8.6
2	6.7	5.1	7.4	6.1	5.4
3	7.9	10.1	9.9	8.9	7.1
4	6.3	7.9	6.3	5.4	5.9
5	11.5	9.1	10.6	9.4	8.5
6	9.6	9.0	9.9	9.7	12.2
7	6.4	6.4	6.6	7.9	6.4
8	7.1	7.4	5.0	5.7	7.1
9	6.7	7.8	7.6	5.8	7.1
10	5.0	5.0	4.4	5.4	6.5
11	8.0	10.6	6.1	6.9	7.2
12	5.4	10.2	6.2	8.5	6.4

Lymphocyte (%) (normal range:27-47)

No	Baseline	1W	1M	3M	9M
1	25.0	29.0	24.8	25.6	23.3
2	24.2	34.9	33.5	32.4	28.0
3	26.8	34.3	35.5	37.8	24.6
4	32.5	28.4	34.8	23.3	31.7
5	36.2	29.7	35.9	32.1	28.8
6	30.2	36.3	30.5	38.6	38.2
7	19.0	15.2	16.0	18.4	19.1
8	46.1	42.9	33.0	37.6	35.4
9	34.9	34.6	33.2	29.9	32.1
10	22.0	18.5	19.9	28.5	25.0
11	28.5	24.0	32.0	25.6	25.9
12	36.7	38.8	35.3	28.6	41.2

Hematocrit (%) (normal range: Male 40-48 Female 34-42)

No	Baseline	1W	1M	3M	9M
1	40.5	39.3	38.2	42.1	36.3
2	38.9	38.6	39.9	40.1	39.0
3	47.7	47.3	46.7	46.0	49.0
4	41.4	40.3	40.4	41.8	41.3
5	42.5	43.4	43.7	43.3	44.4
6	46.3	47.8	47.9	39.9	46.5
7	40.4	40.7	41.3	39.5	41.0
8	38.2	39.9	40.1	39.0	38.8
9	43.1	43.4	42.9	43.0	44.7
10	39.2	39.3	40.0	39.2	41.0
11	36.0	36.6	37.1	36.3	38.0
12	41.6	41.2	39.1	40.7	40.8

Hemoglobin (g/dL) (normal range: Male 13-17 Female 12-16)

No	Baseline	1W	1M	3M	9M
1	13.6	12.9	12.6	14.1	11.7
2	13.1	13.0	13.3	13.5	13.0
3	15.8	15.3	15.3	14.8	15.9
4	13.2	13.1	13.0	13.7	13.3
5	14.4	14.8	14.7	14.7	15.4
6	15.2	15.5	15.9	13.1	15.0
7	12.8	13.0	13.2	12.8	13.3
8	12.4	12.6	12.8	12.8	12.7
9	13.9	14.2	13.9	13.7	15.5
10	13.1	12.7	12.8	13.0	13.6
11	12.1	11.9	12.0	12.1	12.5
12	13.8	13.6	13.4	14.0	14.1

Leucocyte ($10^3/\mu L$) (normal range:4-9)

No	Baseline	1W	1M	3M	9M
1	6.9	6.0	7.1	7.8	7.3
2	6.5	5.0	5.1	6.4	6.3
3	5.7	4.8	5.1	5.2	5.8
4	5.6	4.8	4.9	6.7	5.3
5	6.4	6.8	6.7	8.3	6.2
6	5.3	5.4	5.9	5.8	4.2
7	5.5	5.8	8.0	6.1	6.6
8	3.6	4.3	4.3	4.0	4.9
9	4.3	4.9	5.3	5.6	6.1
10	7.7	5.9	6.9	7.2	6.6
11	5.6	5.0	5.2	6.8	6.4
12	4.5	4.9	5.5	5.6	5.5

Erythrocyte (10^6/µL) (normal range: Male 4.0-5.3   Female 3.8-4.8)

No	Baseline	1W	1M	3M	9M
1	4.68	4.48	4.44	4.83	4.25
2	4.38	4.32	4.51	4.54	4.42
3	5.18	5.04	5.10	4.97	5.27
4	4.55	4.35	4.42	4.59	4.53
5	4.56	4.73	4.72	4.68	4.82
6	5.09	5.24	5.32	4.44	5.07
7	4.27	4.31	4.34	4.17	4.32
8	4.29	4.42	4.49	4.33	4.32
9	4.65	4.74	4.67	4.59	5.00
10	4.13	3.95	4.04	3.96	4.15
11	3.90	4.00	4.01	3.88	4.09
12	4.56	4.52	4.29	4.52	4.57

Albumin (g/dL) (normal range: 4.1-5.1)

No	Baseline	1W	1M	3M	9M
1	4.3	4.4	4.2	4.5	4.0
2	4.4	4.3	4.4	4.6	4.2
3	4.2	3.9	4.2	4.0	4.4
4	4.4	4.3	4.4	4.4	4.5
5	4.0	4.3	4.2	4.2	4.3
6	4.7	4.9	5.0	4.4	4.7
7	4.6	4.5	4.7	4.6	4.6
8	4.3	4.2	4.4	4.3	4.2
9	4.8	5.1	4.6	4.3	4.8
10	4.4	4.1	4.3	4.3	4.6
11	4.2	3.9	4.1	4.0	4.2
12	4.2	4.1	4.2	4.2	4.4

HbA1c (%) (normal range: 4.6-6.2)

No	Baseline	1W	1M	3M	9M
1	5.3	5.3	5.2	5.4	5.4
2	5.3	5.5	5.3	5.5	4.9
3	7.0 a	7.2	7.1	6.7	6.3
4	5.6	5.5	5.7	6.0	6.0
5	5.6	5.6	5.6	5.6	5.6
6	5.3	5.5	5.5	5.8	5.7
7	5.2	5.2	5.2	5.2	5.1
8	5.6	5.6	5.5	5.9	5.5
9	5.0	5.1	5.2	4.9	5.2
10	5.2	5.3	5.2	5.3	4.9
11	5.5	5.5	5.6	5.5	5.4
12	5.0	5.1	5.1	4.9	4.9

a: HbA1c of patient 3 at screening was 6.4%

Glucose (mg/dL) (normal range: 73-109)

No	Baseline	1W	1M	3M	9M
1	84	94	89	90	95
2	94	91	93	95	93
3	127	143	126	114	123
4	101	107	111	97	106
5	81	95	96	90	90
6	96	115	106	90	100
7	93	70	100	90	101
8	97	88	97	97	97
9	90	93	104	93	96
10	107	100	98	95	97
11	86	74	75	85	75
12	95	104	96	99	98

Total Protein (g/dL) (normal range: 6.6-8.1)

No	Baseline	1W	1M	3M	9M
1	7.7	7.6	7.3	7.9	7.6
2	7.2	7.1	7.1	7.4	6.7
3	7.2	7.0	7.2	7.0	7.8
4	7.0	6.8	7.0	7.6	7.3
5	6.4	6.9	6.8	6.8	6.8
6	7.9	8.2	8.2	7.3	8.2
7	7.6	7.3	7.4	7.4	7.3
8	7.1	7.2	7.3	7.2	7.0
9	7.2	7.7	6.8	6.7	7.5
10	7.3	7.1	7.2	7.4	7.6
11	6.8	6.7	6.9	6.7	7.0
12	7.2	7.1	6.9	7.0	7.3

Total bilirubin (mg/dL) (normal range: 0.4-1.5)

No	Baseline	1W	1M	3M	9M
1	0.6	0.6	0.6	0.7	0.7
2	0.6	0.8	0.9	1.0	0.8
3	1.3	1.2	2.0	1.1	1.4
4	0.7	0.8	0.8	0.8	0.6
5	0.8	0.7	0.7	0.8	0.8
6	0.7	0.9	0.7	0.7	0.4
7	0.8	0.5	0.8	0.6	0.7
8	0.6	0.4	0.5	0.8	0.6
9	1.3	1.4	1.3	0.9	1.8
10	0.8	0.4	0.6	0.7	0.7
11	0.6	0.6	0.6	0.8	1.2
12	0.7	0.8	1.0	1.1	0.7

Total cholesterol (mg/dL) (normal range: 142-248)

No	Baseline	1W	1M	3M	9M
1	267	252	263	297	272
2	222	235	240	246	225
3	150	140	158	115	133
4	201	207	212	233	220
5	116	113	117	122	130
6	287	291	301	265	252
7	163	147	163	146	145
8	170	164	174	175	172
9	238	243	217	205	241
10	191	167	177	182	179
11	225	210	224	213	218
12	151	164	166	167	172

LDH (U/L) (normal range: 124-222)

No	Baseline	1W	1M	3M	9M
1	207	210	214	209	193
2	184	180	189	179	184
3	214	204	202	222	192
4	199	217	193	208	203
5	161	180	177	170	182
6	168	173	190	162	181
7	159	148	164	150	164
8	210	200	222	211	202
9	177	175	160	173	182
10	215	206	196	187	211
11	143	122	137	125	135
12	154	154	145	142	154

ALP (U/L) (normal range: 106-322)

No	Baseline	1W	1M	3M	9M
1	293	310	294	255	280
2	217	200	209	205	194
3	274	303	271	248	259
4	204	194	206	223	180
5	158	202	257	188	216
6	141	156	157	122	155
7	153	186	160	161	159
8	225	283	296	234	241
9	103	106	107	104	103
10	249	280	269	264	285
11	148	163	168	139	125
12	134	137	124	123	135

CRP (mg/dL) (normal range: ≤0.14)

No	Baseline	1W	1M	3M	9M
1	0.49	0.42	0.37	0.43	4.85
2	0.19	0.15	0.14	0.14	0.14
3	0.14	0.34	0.14	0.14	0.20
4	0.14	0.16	0.14	0.14	0.14
5	0.14	0.14	0.14	0.14	0.14
6	0.14	0.14	0.14	0.14	0.56
7	0.14	0.14	0.14	0.14	0.14
8	0.14	0.14	0.14	0.14	0.14
9	0.14	0.14	0.14	0.14	0.14
10	0.14	0.46	0.14	0.14	0.14
11	0.14	0.14	0.14	0.14	0.14
12	0.14	0.14	0.14	0.14	0.14

Na (mEq/L) (normal range: 138-145)

No	Baseline	1W	1M	3M	9M
1	143	142	140	141	139
2	143	140	140	141	140
3	142	143	141	143	143
4	142	143	141	143	142
5	143	142	141	138	141
6	141	141	142	143	140
7	141	139	138	140	142
8	142	144	145	145	143
9	142	142	143	142	141
10	144	143	143	146	143
11	140	140	144	141	141
12	144	142	142	139	142

K (mEq/L) (normal range: 3.6-4.8)

No	Baseline	1W	1M	3M	9M
1	3.7	3.7	4.1	4.0	4.0
2	4.2	4.4	3.9	3.8	4.0
3	4.2	4.4	4.3	4.2	4.2
4	3.8	4.0	3.9	4.1	4.2
5	4.4	4.5	4.7	4.0	4.4
6	4.0	3.8	4.1	3.7	4.2
7	3.6	3.6	3.7	3.6	3.9
8	4.9	4.8	4.1	3.6	4.1
9	4.0	4.0	4.3	4.4	4.4
10	3.8	4.2	3.9	4.0	4.3
11	4.1	4.0	3.9	4.1	3.9
12	4.2	3.7	3.9	4.2	3.9

Cl (mEq/L) (normal range: 101-108)

No	Baseline	1W	1M	3M	9M
1	104	104	104	102	105
2	107	105	104	105	106
3	107	108	106	110	105
4	104	105	103	104	106
5	104	104	104	101	105
6	104	101	104	105	101
7	104	103	103	105	105
8	107	108	110	107	109
9	105	105	105	105	105
10	106	106	106	108	106
11	103	104	107	104	104
12	107	105	106	105	107

BUN (mg/dL) (normal range: 8.0-20.0)

No	Baseline	1W	1M	3M	9M
1	10.6	10.6	14.0	9.8	12.6
2	11.4	8.2	9.7	10.7	11.6
3	9.9	13.2	13.0	11.2	14.7
4	15.2	20.0	18.4	18.0	15.5
5	14.2	13.5	14.5	12.3	17.4
6	10.0	11.8	12.1	10.3	11.3
7	14.9	15.8	11.2	15.9	13.5
8	17.6	21.2	15.7	16.1	15.8
9	13.7	14.9	12.5	14.1	13.8
10	15.0	15.5	15.3	15.1	16.3
11	18.6	18.4	18.6	17.9	16.5
12	9.3	8.2	10.8	9.9	10.1

Creatinine (mg/dL) (normal range: Male 0.65-1.07 Female 0.46-0.79)

No	Baseline	1W	1M	3M	9M
1	0.6	0.7	0.7	0.8	0.7
2	0.6	0.6	0.6	0.5	0.5
3	1.0	1.1	1.1	1.0	1.1
4	0.7	0.7	0.7	0.7	0.8
5	0.9	0.9	0.9	0.9	1.0
6	0.5	0.5	0.5	0.6	0.6
7	0.5	0.6	0.5	0.6	0.6
8	0.7	0.6	0.7	0.7	0.6
9	0.5	0.6	0.5	0.5	0.6
10	0.7	0.7	0.7	0.7	0.8
11	0.6	0.6	0.6	0.6	0.6
12	0.6	0.6	0.6	0.6	0.6

Uric acid (mg/dL) (normal range: Male 3.7-7.8 Female 2.6-5.5)

No	Baseline	1W	1M	3M	9M
1	6.3	6.1	6.6	6.5	6.8
2	3.4	3.6	3.5	3.2	3.4
3	5.2	5.5	5.9	5.2	5.4
4	4.2	4.3	4.1	4.2	3.9
5	7.9	7.2	8.2	7.2	8.7
6	4.8	5.4	4.5	4.7	4.3
7	3.9	4.7	4.0	4.6	4.6
8	5.3	4.7	4.7	4.6	4.1
9	2.4	2.9	2.9	2.8	2.7
10	4.3	3.9	4.8	4.2	4.9
11	3.8	3.4	3.2	3.8	3.4
12	3.7	3.8	3.8	3.8	4.2

AST (U/L) (normal range: 13-30)

No	Baseline	1W	1M	3M	9M
1	22	38	25	21	31
2	14	15	15	15	14
3	53	48	43	36	31
4	31	26	26	27	23
5	34	29	29	30	31
6	25	25	26	21	29
7	20	19	20	22	21
8	20	19	22	24	18
9	14	16	12	10	12
10	31	29	29	23	23
11	15	14	14	12	14
12	18	17	15	15	17

ALT (U/L) (normal range: Male10-42 Female 7-23)

No	Baseline	1W	1M	3M	9M
1	27	37	36	19	34
2	9	11	11	11	9
3	84	71	63	47	39
4	41	26	26	25	22
5	37	33	31	33	29
6	23	24	32	21	23
7	11	10	10	11	11
8	12	12	13	12	12
9	11	10	10	10	9
10	28	33	26	21	20
11	15	14	14	11	14
12	11	11	10	9	12

CPK (U/L) (normal range: Male 59-248 Female 41-153)

No	Baseline	1W	1M	3M	9M
1	92	97	116	92	79
2	77	81	103	79	65
3	98	93	114	105	154
4	110	163	101	93	112
5	266	263	179	245	209
6	80	94	102	64	79
7	91	86	103	88	98
8	112	130	136	116	118
9	64	82	59	65	63
10	269	196	160	116	168
11	70	57	85	46	62
12	53	44	45	43	50

Supplemental table 4. Individual data of PD reduction, CAL gain and new bone formation.

	PD reduction (mm)			CAL gain (mm)			new bone formation (%)			
No	3M	6M	9M	3M	6M	9M	1M	3M	6M	9M
1	3	4	4	2	2	2	22.94	38.21	49.64	107.96
2	5	5	5	4	4	4	21.03	21.37	20.27	21.00
3	3	3	3	2	2	2	12.51	31.09	51.89	75.55
4	3	3	3	3	3	3	2.05	16.09	26.83	33.61
5	3	3	3	3	3	3	1.13	49.58	73.67	84.72
6	3	4	4	3	5	5	-15.31	6.98	29.45	73.68
7	3	3	3	3	2	2	24.41	13.82	19.56	11.88
8	4	5	5	4	4	4	0.00	24.65	72.65	74.05
9	2	2	2	1	1	1	8.24	29.16	27.88	36.93
10	2	2	3	1	1	2	15.77	16.37	-4.57	22.80
11	4	8	8	3	7	7	11.65	21.48	21.84	32.81
12	0	1	1	0	1	1	2.69	6.98	11.24	14.54
